# SNPs in 3′UTR miRNA Target Sequences Associated with Individual Drug Susceptibility

**DOI:** 10.3390/ijms232213725

**Published:** 2022-11-08

**Authors:** Elena Rykova, Nikita Ershov, Igor Damarov, Tatiana Merkulova

**Affiliations:** 1 Institute of Cytology and Genetics, Siberian Branch of Russian Academy of Sciences, 630090 Novosibirsk, Russia; 2 Institute of Chemical Biology and Fundamental Medicine, Siberian Branch of Russian Academy of Sciences, 630090 Novosibirsk, Russia; 3 Department of Engineering Problems of Ecology, Novosibirsk State Technical University, 630087 Novosibirsk, Russia

**Keywords:** microRNA (miRNA), miRNA target sites, single nucleotide polymorphisms (SNPs), 3′untranslated regions (3′UTRs), drug response, pharmacogenes, luciferase reporter assay, PASSPORT-seq, allele-specific expression (ASE), *VEGFR1*

## Abstract

The complementary interaction of microRNAs (miRNAs) with their binding sites in the 3′untranslated regions (3′UTRs) of target gene mRNAs represses translation, playing a leading role in gene expression control. MiRNA recognition elements (MREs) in the 3′UTRs of genes often contain single nucleotide polymorphisms (SNPs), which can change the binding affinity for target miRNAs leading to dysregulated gene expression. Accumulated data suggest that these SNPs can be associated with various human pathologies (cancer, diabetes, neuropsychiatric disorders, and cardiovascular diseases) by disturbing the interaction of miRNAs with their MREs located in mRNA 3′UTRs. Numerous data show the role of SNPs in 3′UTR MREs in individual drug susceptibility and drug resistance mechanisms. In this review, we brief the data on such SNPs focusing on the most rigorously proven cases. Some SNPs belong to conventional genes from the drug-metabolizing system (in particular, the genes coding for cytochromes P450 (CYP 450), phase II enzymes (SULT1A1 and UGT1A), and ABCB3 transporter and their expression regulators (PXR and GATA4)). Other examples of SNPs are related to the genes involved in DNA repair, RNA editing, and specific drug metabolisms. We discuss the gene-by-gene studies and genome-wide approaches utilized or potentially utilizable to detect the MRE SNPs associated with individual response to drugs.

## 1. Introduction

Recently, large scale sequencing and genome-wide association studies (GWASs) have identified tens of thousands of variants (mainly, single nucleotide polymorphisms, SNPs) associated with various human traits and diseases [1,2,3]. Of them, nearly 90% are located in noncoding regions [4,5] and can impact gene expression in several ways. A large group of the SNPs mainly residing in promoter and enhancer regions influences transcription initiation by creating or disrupting transcription factor binding sites or changing the affinity of transcription factors for their cognate sites [6,7,8,9]. Many SNPs affect RNA splicing [6,10]. In addition, 3′untranslated regions (3′UTRs) of genes often contain the variants which are able to change the binding affinity of targeting miRNAs, thereby interfering with gene expression at a posttranscriptional level [11,12].

According to the current data, miRNAs interact in a complementary manner with their sites in the 3′UTRs of the mRNAs of target genes causing template degradation and translational repression, thus playing a leading role in the posttranscriptional mechanisms of gene expression regulation [13]. MicroRNAs (miRNAs), an evolutionarily conserved class of endogenous 18–24-nucleotide noncoding RNAs, form a RISC complex together with one of the AGO family proteins and auxiliary proteins and are involved in translation repression or cleavage of target mRNAs [14,15]. So far, over 2600 miRNAs have been identified in humans [16]. One miRNA has the potential to target many genes (on the average, approximately 500 genes) and, according to the estimates, over 60% of the human mRNAs are potential targets of miRNAs [17].

Currently, a significant amount of data suggest that the coordinated action of miRNAs plays an important role in the regulation of biological processes, including the cell cycle, cell growth and differentiation, migration, apoptosis, and stress response [18]. Numerous studies have also shown that an altered expression of miRNAs contributes to the development of a wide range of human pathologies, such as cancer, diabetes, neuropsychiatric disorders, cardiovascular diseases, and infection diseases [19,20,21,22,23]. The SNPs interfering with the interaction of miRNAs with their target sites, located in both miRNA seed sequences and mRNA 3′UTRs, are frequently associated with the same diseases (for review, see [12]). In addition, some data suggest the involvement of SNPs in 3′UTR miRNA target sequences in individual drug susceptibility and drug resistance mechanisms [24,25,26]. In this review, we attempt to describe and systematize the information about such SNPs accumulated so far focusing on the most reliably proved relevant cases and the methods used for this purpose. We also describe and discuss the state-of-the-art genome-wide approaches utilized or potentially utilizable to detect the SNPs influencing the interaction between miRNAs and their target sites and associated with individual drug response.

## 2. Modern Approaches to Identify Functional SNPs in 3′UTR miRNA Target Sequences

**Figure 1:**
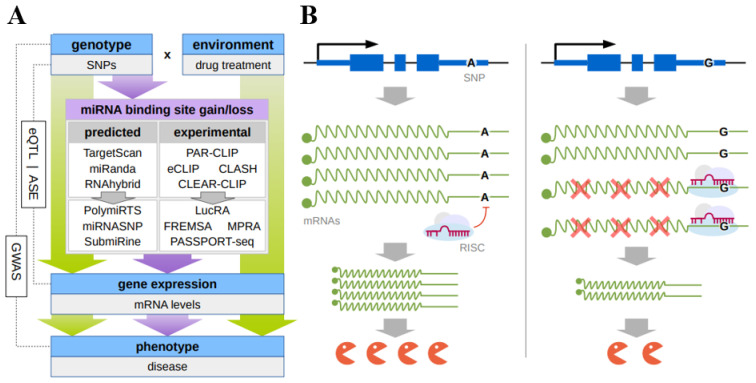
(**A**) Principal scheme of discovery of functional SNPs that result in changes of microRNA binding to their response elements in the mRNA 3’UTR; (**B**) the hypothetical mechanism of the individual drug response due to the mirSNP. SNP(A>G) in 3′UTR of mRNA leads to the gain of the miRNA response element allowing miRNA-mRNA binding which hinders gene expression at a posttranscriptional level.

An integrated set of in silico, in vitro, and in vivo approaches has been designed so far to detect the SNPs located in mRNA 3′UTRs and influencing (both deteriorating and enhancing) the interaction of miRNAs with their target sites (Figure 1A).

As a rule, in silico approaches rely on a certain algorithm predicting miRNA binding sites that make it possible to assess the functionality of these sites or correspondingly rank them based on the type of seed match, site accessibility, evolutionary conservation, nucleotide environment, thermodynamic characteristics of the duplex, and so on. In particular, the databases PolymiRTS 3.0 (http://www.targetscan.org/vert_71/) (accessed on 10 October 2022) and miRNASNP-v3 (http://bioinfo.bjmu.edu.cn/mirsnp/search/) (accessed on 10 October 2022) or the tool SubmiRine (https://research.nhgri.nih.gov/software/SubmiRine/) (accessed on 10 October 2022) use the popular de novo prediction algorithm TargetScan (http://www.targetscan.org/vert_71/) (accessed on 10 October 2022). This algorithm utilizes the so-called context+ score regression model to assess the site efficiency, which takes into account the contribution of the parameters, such as seed-pairing stability, target-site abundance, local AU content, site location within 3′UTR, and 3′-supplementary pairing (and a more recent context++ model, based on 14 parameters, including the degree of site conservation). Thus, the SNPs are selected that either differ in their context+ score or absent in one of the allelic variants. An alternative in silico approach used, in particular, in miRNASNP-v3, as well as popular and effective in silico method to study individual sites is the calculation of the minimum free energy (MFE) of hybridization between a miRNA seed and its cognate mRNA target (for example, using MicroInspector, RNAfold and RNAhybrid) [27,28,29].

A set of experimental approaches allowing for gene-by-gene or genome-wide studies is used to verify the obtained predictions. The main in vitro gene-by-gene approaches are luciferase reporter assay and fluorescent-based RNA electrophoretic mobility shift assay (FREMSA or simply RNA EMSA).

The luciferase reporter assay is used in almost all studies on assessment of the effect of a nucleotide substitution on the efficiency of miRNA–mRNA interaction. This method utilizes the plasmid constructs carrying a mutated or a reference allele of miRNA recognition site located in the mRNA 3′UTR inserted downstream of the reporter luciferase gene. Each plasmid construct is transfected alone [30] or cotransfected with the miRNA into the culture of selected eukaryotic cells to monitor the reporter expression [31]. Either siRNA overexpressing plasmids [32], miRNA mimics (individually [11,27,28,33] or a library of miRNA mimics [34] are used for cotransfection. Deletion and mutagenesis of the putative miRNA response element (MRE) in the plasmid construct are used to confirm the location of binding site for each miRNA [35]. Both transient [11,27,28,33] and stable transfection of cultivated cells with the plasmid reporter construct with concurrent cotransfection of mimic miRNA are used [36].

Although many papers assert that the luciferase reporter assay is able to determine the differences in the binding affinities between the wild-type and alternative alleles, this method in fact can record only the suppressive effect of a miRNA on the expression of its putative targets [31,32,37], which only allows the effect of the corresponding nucleotide substitution on the affinity of miRNA–mRNA interaction to be assumed. In addition, a considerable limitation of this method is the tissue-specificity of the studied effects, which requires that several cell lines are used [38].

FREMSA is an in vitro technique which is able to provide direct evidence of the interaction between miRNA and mRNA and to assess the effect of an SNP on the efficiency of this interaction [27,39,40]. This method utilizes the RNA oligonucleotides corresponding to the mature miRNA species and the cognate mRNA fragments corresponding to the miRNA targeting sequences. The fragments are 5′-labeled with different fluorescent dyes. The reaction mixture is separated by gel electrophoresis; the bands representing miRNAs, mRNAs, and miRNA–mRNA complexes are visualized according to different wavelengths of fluorescence dyes [27,39,40].

Correlation analysis of the expression of the miRNA and mRNA carrying different alleles in tissue samples is an informative in vivo method, widely used to assess the potential regulatory effects of miRNAs on their targets [32,34,41]. Another approach utilizes the transient transfection of primary human cells with miRNA mimics or the miRNA mimic negative controls in order to evaluate their influence on the target enzyme activity or mRNA concentrations [35]. The miRNA inhibitors, leading to a decrease in their levels in cells and the coupled decrease in the levels of mRNA targets, are also used [28].

To date, over 400,000 SNPs have been identified in putative miRNA binding sites (mirSNPs) by in silico methods, which poses the challenge of designing the genome-wide approaches to search for functional SNPs affecting miRNA binding [38]. The advent of state-of-the-art genome-wide technologies has brought about some such methods. In particular, the MPRA (massive parallel reporter analysis) [42] made it possible in 2018 to develop the PASSPORT-seq (parallel assessment of polymorphisms in miRNA target sites by sequencing) approach, which involves pooled synthesis, parallel cloning, and single-well transfection followed by next-generation sequencing (NGS) to functionally test hundreds of mirSNPs at once [38]. First, the utility of the assay was demonstrated by study of 100 randomly selected mirSNPs in HEK293, HepG2, and HeLa cells. Using traditional individual luciferase assay, a gold-standard platform for in vitro assessing gene expression, 17 of the tested 21 PASSPORT-seq results were confirmed. Second, the application of PASSPORT-seq technology to 111 mirSNPs in 3′UTR of 17 pharmacogenes showed a significant effect of 33 variants in at least one cell line (HeLa, HepG2, HEK293, or HepaRG). Several examples from this list are included below.

NGS was also used to design AGO crosslinking immunoprecipitation (CLIP) technologies that allow for the identification of endogenously relevant transcriptome-wide sites bound by AGO (eCLIP, PAR-CLIP, and HITS-CLIP). However, these technologies fail to reveal which particular miRNAs bind to each mRNA target [43,44]. A ligation step added to the CLIP technology makes it possible to sequence the chimeric miRNA–mRNA molecules, thereby showing the pairs that have hybridized (crosslinking, ligation, and sequencing of hybrids, CLASH) [45]. Powell et al. [44] used this approach to generate a liver-expressed pharmacogene-relevant map of miRNA–mRNA interactions and target sites. In their study, the chimeric-eCLIP was used to experimentally define the miRNA–mRNA interactome in primary human hepatocytes from a pool of 100 donors. Based on these results, an extensive map of miRNA binding of each gene was developed, including the pharmacogenes expressed in primary hepatocytes. Using the high-throughput PASSPORT-seq assay in HepaRG cells, they further tested the functional impact of 262 genetic variants in these miRNA binding sites. These 262 variants were selected among approximately 3600 PharmGKB variants as having the coordinates overlapping their chimeric-eCLIP–identified target sites. As a result, they discovered 24 mirSNPs considered to be functional in HepaRG cells. The share of the detected functional SNPs in their work is lower as compared with the earlier study of this group reported by Ipe et al. [38] although both studies were carried out using the same approach. The putative cause of this discrepancy is that in the later study Powell et al. used only one cell line versus four lines used in the earlier study by Ipe et al. [44]. Another possible cause is that in the later study [44] the SNPs were involved initially localized not only in 3′UTR, but also in the gene coding regions. In particular, the combination of chimeric-eCLIP with PASSPORT-seq successfully identified the functional mirSNPs at the end of the DPYD pharmacogene coding region that influenced the hsa-mir-27b interaction with the earlier validated binding site [46]. Note that these results suggest a potentially important role of the binding to target sequences not only in the 3′UTR but in the coding regions as well.

Find the examples of application of the described methods in the chapter below.

## 3. Variation in 3′UTR miRNA Target Sequences of the Genes Involved in Drug Metabolism

Phase I and phase II drug metabolizing enzymes, drug transporters (DMETs), and a number of transcription regulators (mainly nuclear receptors, NRs) are involved in the metabolism and excretion of xeno- and endobiotics and, therefore, play an important role in overall response to drugs [47]. The hereditary variation of the drug transformation can result from the presence of polymorphisms in the related genes, mainly SNPs, which are able to modify coding or regulatory sequences. Changes in DMETs and the activity of their transcription regulators may lead to variable levels of a drug and, consequently, have significant impact on the drug efficiency and safety, potentially disrupting pharmacological effects or causing adverse events. Therefore, the search for the inter-subject variability in pharmacokinetics and drug response has been mainly focused on this system [48]. Correspondingly, the overwhelming majority of the known examples of the SNPs in the 3′UTRs that affect the miRNA binding and, as a consequence, the mRNA level are related to the genes belonging to this system (Figure 1B). 

### 3.1. Phase I Enzymes

#### 3.1.1. CYP2E1

The first study to show that SNPs in the 3′UTR influence the miRNA-dependent regulation of cytochromes P450 was the targeted study of cytochrome P450 2E1 (CYP2E1), highly expressed in the human liver [36]. CYP2E1 catalyzes the metabolism of a variety of important endogenous biochemicals and xenobiotics, as well as approximately 3% of the commercially available drugs. The team of Nakajima studied the possibility that miRNAs were involved in the SNP-mediated modulation of *CYP2E1* expression and discovered that two neighboring SNPs in the 3′UTR of *CYP2E1* 1556T>A = rs2480257 and 1561A>G = rs2480256 destroyed the binding site of miR-570 [36]. First, a computational search using MicroSNiPer (http://epicenter.ie-freiburg.mpg.de/services/microsniper/) (accessed on 10 October 2022) predicted that miR-570 bound to the sequence around the SNPs. The miR-570 recognition element (MRE) is located closely upstream of the MRE of miR-378, which was earlier found to regulate *CYP2E1* expression [49]. Then, a luciferase assay using transient expression of the reporter plasmids containing the fragments with the MRE of haplotype TA or AG was performed with HEK293 cells. The cotransfection with the miR-570 mimic suggested that miR-570 recognized the MRE of the *CYP2E1* haplotype TA. They further constructed the HEK293 cell lines stably expressing haplotype TA or haplotype AG and transfected miR-570 and miR-378 mimics into HEK/2E1(TA) and HEK/2E1(AG). When miR-570 was transfected into HEK/2E1(TA) cells, the CYP2E1 protein level significantly decreased (to 60% of the control); however, this was unobservable in the HEK/2E1(AG) cells. The overexpression of miR-378 declined the *CYP2E1* expression levels in both HEK/2E1(TA) and HEK/2E1(AG) cells. These results indicate that miR-570 and miR-378 regulate *CYP2E1* expression in haplotype-dependent and haplotype-independent manners, respectively. In 32 human liver specimens, CYP2E1 levels show no positive correlation with *CYP2E1* mRNA levels, suggesting that posttranscriptional regulation plays an important role for CYP2E1. In seven human livers with diplotype TA/TA, the CYP2E1 levels inversely correlate with the miR-570 levels but any correlation is unobservable in 25 human livers with diplotypes TA/AG and AG/AG [36]. PASSPORT-seq results support the above described data [38]. A significant change in the variant expression was demonstrated for both neighboring SNPs, especially for rs2480256: the expression of allele G significantly increased in all tested cell lines. In a recent study, Maillard et al. [50] described the association of rs2480256 with the chemotherapy-induced hepatotoxicity in patients suffering from advanced soft tissue sarcoma. Analysis of the trabectedin-treated patients with isolated elevation of γ-GT blood concentration demonstrated the rs2480256 variant of the *CYP2E1* gene to be significant [50].

#### 3.1.2. CYP3A5

CYP3A5 is a member of CYP3A family, involved in the metabolism of many compounds. Myasthenia gravis is an autoimmune neuromuscular junction disease, currently treated with new efficient immunosuppressive drugs, for example, tacrolimus. However, the high inter-subject variation in tacrolimus metabolism in liver severely limits its use in clinical practice. As is known, CYP3A4 and CYP3A5 are the main tacrolimus metabolizing enzymes [51]. To search for genetic factors involved in the clinical response to tacrolimus therapies in Myasthenia gravis patients, this team screened 86 patients treated with tacrolimus for clinical features as well as genetic data [51]. As a result, six SNPs, including one in *CYP3A5* (rs15524), potentially related to blood levels of the drug in serum were found. Then, using the MirSNP and SNPinfo (https://snpinfo.niehs.nih.gov/) (accessed on 10 October 2022) databases the authors found that rs15524 affect potential hsa-miR-500 binding site in 3′UTR of *CYP3A5* gene. Using the dual-luciferase reporter assay in HepG2 cells they proved that the inhibitory effect of exogenously transfected hsa-miR-500a mimics on the expression of the reporter gene was much stronger in the case of the T allele compared to C allele [51]. These results could explain the downregulated *CYP3A5* expression (in the miR-500–overexpression cell lines) and a higher tacrolimus serum concentration in the wild-type T allele rs15524 carriers [52]. PASSPORT-seq showed an increased expression of rs15524 *CYP3A5* variant C allele as compared with the reference T allele in four tested cell lines, matching the results of luciferase reporter assay by [38,51]. Another study demonstrated that *CYP3A5* rs15524 had a certain influence on the concentration of another immunosuppressive drug, sirolimus, in the blood of kidney-transplanted patients [53].

#### 3.1.3. CYP2B6

CYP2B6 is one of the minor CYPs in the human liver. Currently, CYP2B6 is estimated to metabolize, fully or partially, approximately 8–13% of the clinically important drugs and other xenobiotics of toxicological relevance. A number of these drugs include efavirenz and nevirapine, used in the treatment of HIV/AIDS, as well as methadone, bupropion, artemisinin, and ketamine. *CYP2B6* expression is highly variable due to extensive genetic polymorphism and inducibility. *CYP2B6* mRNA has a long (1569 bp) 3′UTR, which is the main target region for miRNAs. Many miRNAs were in silico predicted to bind to the 3′UTR of *CYP2B6* using different algorithms and databases. Burgess et al. [54] studied genetic variants in the *CYP2B6* 3′UTR of 200 healthy volunteers that were administered efavirenz, which was shown to be a suitable probe drug substrate to measure CYP2B6 activity in vitro and in vivo. In total, 90 human liver microsomal preparations isolated from liver tissue samples were sequenced and characterized for CYP2B6 activity by measuring bupropion hydroxylation. They reported that dinucleotide polymorphism rs70950385 (AG>CA), being a combination of rs12979270 (A>C) and rs12979898 (G>A) variant alleles, was associated with decreased efavirenz and bupropion hydroxylation activities in microsomes of the genotyped human liver specimens [54]. For the rs70950385 dinucleotide variant, manual seed sequence alignment was performed to identify the miRNA binding sites. The mutant rs70950385 (CA) variant, predicted to create a binding site for miR-1275, was associated with a 33% decrease in the CYP2B6 activity among normal metabolizers. In vitro luciferase assays in HepG2 cells were used to confirm that the variant CA allele created a miRNA binding site, causing an 11% decrease in the luciferase activity as compared with the wild-type AG allele when cotransfected with miR-1275. The results show that a 3′UTR mirSNP contributes to the variation in CYP2B6 efavirenz metabolizing activity. The effect of *CYP2B6* genetic variant rs12979270 (A>C), which is a part of dinucleotide rs70950385, was shown to be statistically significant in HepaRG cells with PASSPORT-seq assay [38]. A statistically significant decrease in the expression in the case of the C allele was observed, which agrees with the effect of the rs70950385 (CA) dinucleotide variant allele in luciferase assay according to the data briefed above [54].

Recently, *CYP2B6* rs3181842 was selected in a combination of 10 SNPs able to construct the SNP-based formula, which was used to calculate the concentration of the intravenous sedative propofol for patients under surgery [30,55] reported that the *CYP2B6* rs3181842 reference C allele functionally led to a low *CYP2B6* expression and activity, resulting in a low metabolic capacity for the formation of dichlorodiphenyldichloroethylene (DDE) from dichlorodiphenyltrichloroethane (DDT) insecticide. The authors used luciferase transfection into HepG2 and LO2 cells and found that the rs3181842 C allele decreased the luciferase expression by 72 and 66% in a statistically significant manner as compared with the T allele in HepG2 and LO2 cells, respectively. They transfected the *CYP2B6*-T/C carrying plasmids into 7901 cells in the absence of *CYP2B6*. A significant decrease in CYP2B6 activity and protein expression was observed in rs3181842 C as compared with the T allele. Additionally, Ipe et al. [38] reported that the rs3181842 C allele created miR-1581/4537 binding sites, absent in the T allele, indicating the possible role of miRNA in the CYP2B6 DDT metabolizing activity.

Other mirSNPs in *CYP2B6* 3′UTR positive in PASSPORT-seq associated with drug metabolism and drug effects were recently found.

*CYP2B6* rs707265 was found to be associated in a statistically significant manner with (S)-methadone plasma levels [56]. Wang et al. [57] examined polymorphisms in the intronic region and 3′UTR of *CYP2B6* and demonstrated that the AGATAA (a combination of rs2279342, rs3745274, rs2279343, rs2279345, rs1038376, and rs707265) *CYP2B6* haplotype was significantly correlated with a higher clearance, lower plasma concentration, and lower concentration-to-dosage (C/D) ratio of (S)-methadone. In general, more methadone can be metabolized in the presence of functional enzymes, resulting in a lower plasma concentration [57]. So far, only PASSPORT-seq has been used in the experimental studies of *CYP2B6* rs707265. As is reported [38], the rs707265 G allele creates miR-1623/4269/3622a-5p binding sites, absent in the A allele, suggesting a possible role of miRNA in the CYP2B6 methadone metabolizing activity.

*CYP2B6* rs1038376 (A>G) variant is found to be associated with a greater risk of methadone-induced PONV (postoperative nausea and vomiting) [58]. The *CYP2B6* poor metabolizers (GG) provide an over twofold lower methadone metabolism as compared with normal/rapid metabolizers. The AG/GG variants of rs2279343 SNP displayed 2.86-fold higher incidence of PONV. According to PASSPORT-seq, the A to T substitution in rs1038376 emerged to be significant; moreover, the T allele displays a higher expression in two cell lines, which describes the effect of another substitution variant as compared with the data by Packiasabapathy et al. [38].

#### 3.1.4. CYP3A7

*CYP3A7* is mainly expressed in the fetal life. However, some people express *CYP3A7* mRNA in the adulthood. CYP3A7 has been reported to influence the pharmacokinetics of an immunosuppressant drug, tacrolimus, widely used for prevention of acute rejection following solid organ transplantation [59]. However, tacrolimus has a narrow therapeutic window with large inter- and intra-individual variations in its pharmacokinetics due to SNPs in the *CYP3A5*, *CYP3A4*, and *CYP3A7* [60,61].

In total, 138 liver transplant recipients and matched donors were genotyped for *CYP3A7* (rs10211 T>C and rs2257401), *CYP3A4* (rs4646437 and rs2242480), and CYP3A5*3 (rs776746) polymorphisms [60]. The *CYP3A7* rs10211 AA carriers (186.2 vs. 90.5, *p* < 0.001) showed an almost twofold increase in the tacrolimus C0/D as compared with the noncarriers (AG and GG genotypes) due to a decreased CYP3A7 activity. The authors conclude that the large inter-individual variation in the tacrolimus concentrations at the early stages after liver transplantation is influenced by genetic polymorphisms of *CYP3A7*, *CYP3A4*, and *CYP3A5* [60]. These results comply with the data collected by Sohn et al. [61]. So far, only PASSPORT-seq has been used in corresponding experimental studies. This approach has shown that the C to T substitution (rs10211) generates potential miR-4732-3p, miR-125a-5p/125b-5p/351/670/4319, and miR-3920 binding sites and somewhat decreases the expression of the reporter construct carrying the T variant allele in two of the four studied constructs [38].

### 3.2. Phase II Enzymes

Uridine diphosphate glucuronosyltransferases (UGTs), glutathione S-transferases (GSTs), sulfotransferases (SULTs), N-acetyltransferase (NAT), and thiopurine methyltransferase (TPMT) are the most important phase II enzymes [62].

#### 3.2.1. SULT1A1

Sulfotransferases (SULTs) are involved in the biotransformation of a variety of drugs. By adding a sulfonyl group to xeno- and endobiotics, more hydrophilic molecules are obtained facilitating their renal excretion [63]. SULT1A1 is the most expressed isoform of the SULT enzymes in the human liver [26]. *SULT1A1* is genetically highly variable among different ethnicities and displays the SNPs affecting expression and function [26,64]. Tamoxifen is an endocrine drug that has significantly improved outcomes in estrogen receptor–positive breast cancer. However, a wide inter-variability of the treatment response is observed [65]. In the tamoxifen metabolism, SULT1A1 catalyzes the transformation of the active metabolites 4-hydroxy-tamoxifen and endoxifen into inactive 4-hydroxy-tamoxifen sulfate and endoxifen sulfate [63]. Earlier, Yu et al. [28] discovered two SNPs in the 3′UTR of the *SULT1A1* gene—902A > G (rs6839) and 973C > T (rs1042157)—associated with a decreased SULT1A1 activity. The free energy in silico analyzed by MicroInspector and RNAhybrid programs predicted that miR-631 would downregulate *SULT1A1* at the rs1042157 T allele more strongly as compared with the C allele. In vitro dual-luciferase reporter assays and overexpression of miR-631 using specific miR-631 inhibitor in three breast cell lines confirm that the T allele reduced luciferase activity relative to the C allele in miR-631 in a dose-dependent manner [28]. In PASSPORT-seq, rs1042157 T allele did not show any significant effect as compared with the reference C allele in the studied cell lines. This result is explainable by the fact that different cell lines were used [28,38]. However, PASSPORT-seq demonstrated a significantly decreased expression of the rs6839 variant G allele as compared with the reference A allele in four tested cell lines, agreeing with earlier publications [28,38].

A recent study [63] evaluated the role of rs6839 and rs1042157 on the tamoxifen metabolism and clinical outcome in the patients with breast cancer receiving tamoxifen (667 cases) enrolled in the completed prospective clinical study (CYPTAM study, NTR1509). The aim was to relate the predicted SULT1A1 phenotypes and endoxifen serum concentrations with clinical outcomes. Three following groups were defined as SULT1A1 activity groups: the low activity group included the combination of rs6839 (GG) and rs1042157 (TT); high activity group, rs6839 (AA) and rs1042157 (CC); and the other combinations formed the medium group. The low SULT1A1 activity group demonstrated higher concentrations of endoxifen and 4-hydroxy-tamoxifen as compared with the medium and high activity groups. In terms of relapse, the low activity group had a better outcome as compared with the medium and high SULT1A1 activity group [63].

#### 3.2.2. UGT1A

UDP-glucuronosyltransferases (UGTs) are conjugation enzymes that detoxify a wide range of both endogenous and exogenous substrates by glucuronidation of free alcohol and phenol groups. In humans, the functional enzymes are classified into three subfamilies, UGT1A, UGT2A, and UGT2B with considerable genetic variation [66]. All *UGT1A* genesshare a common polymorphic 3′UTR containing three linked SNPs (rs10929303, rs1042640, and rs8330) which are able to influence miRNA binding. Common rs3100 SNP is also present within the 3′UTR of *UGT2B15*, coding for one of the seven functional enzymes of the UGT2B subfamily. A posttranscriptional repression of *UGT1A*, *UGT2B7*, and *UGT2B15* expression by miRNAs is regarded as an important mechanism underlying the inter-individual variability in drug glucuronidation. Papageorgiou and Court [34] aimed to identify the miRNAs that could regulate *UGT1A*, *UGT2B7*, and *UGT2B15* expression through binding to the variant 3′UTRs. The luciferase reporter plasmids containing either the reference or variant 3′UTR were screened against a 2048 human mimic miRNA library to identify the miRNAs that decreased the luciferase activity by at least 30% when cotransfected into HEK293 cells [34]. Using the miRanda and RNAhybrid programs, they identified the miRNAs that repressed either *UGT1A* or *UGT2B* 3′UTR with several of them selectively repressing either the reference or variant allele in individual luciferase reporter assays in HEK293 cells. The repression was abrogated when the corresponding MRE was deleted in the plasmid constructs. In particular, the rs8330 variant C allele disrupted the miR-1286 binding and the G allele did not. Moreover, miR-21-3p MRE was created by the rs10929303 T>C SNP in the *UGT1A* variant C allele. The effect of the rs8330 G>C nucleotide substitution on the miR-1286 MRE was further confirmed by evaluating the luciferase constructs carrying the reference sequence (G allele) of the miR-1286 MRE triplicated in forward and reverse orientations. The transfection of miR-21-3p, miR-141-3p, and miR-200a-3p into primary human hepatocytes repressed both the UGT1A1 activity and mRNA without affecting the CYP3A activity. Finally, miR-21-3p and miR-200a-3p expression negatively correlated with the UGT1A6 activity and mRNA in human liver samples [35]. PASSPORT-seq showed an increased expression of the rs8330 variant C allele as compared with the reference G allele in HeLa cell line [38]. According to PASSPORT-seq, the rs10929303 variant C allele also demonstrated an increased gene expression as compared with the reference T allele in HepaRG cell line [38].

The *UGT1A1* phenotypes coded for by alleles C/C for both SNPs (rs8330 and rs10929303) are categorized as fast glucuronidators based on their plasma acetaminophen glucuronidation status [67]. Earlier, the association of rs8330 with a higher acetaminophen glucuronidation activity in liver samples and a decreased risk of unintentional acetaminophen-induced liver failure was reported [68]. Recently, a number of pharmacogene SNPs have been studied in association with the gefitinib-induced rash, mainly featured by papulopustular rash, which is the most common adverse reaction to gefitinib anticancer therapy. The variant allele rs10929303 of *UGT1A* correlates with gefitinib plasma exposure since the concentration of gefitinib in the non-small cell lung cancer patients with TT genotypes is significantly lower as compared with any other genotypes [69]. Together, these studies open up a variety of interesting pharmacogenetic possibilities on the polymorphic regulation of glucuronidation pathways through mirSNPs.

### 3.3. Transporters

The ATP-binding cassette transporters (ABC transporters) are a family of membrane proteins that transport a wide range of substrates, including metabolic products, nutrients, lipids, and drugs, across the extracellular and intracellular membranes. The ABC transporters are involved in xenobiotic metabolism and the alterations in their expression can result in important impacts on human health. Phylogenetic analysis groups the 49 known human ABC transporters into 8 distinct subfamilies (ABCA–ABCH) [70].

The overexpression of ABC subfamily B member 3 (ABCB3), or the transporter associated with antigen processing 2 (TAP2), is associated with multidrug resistance to anticancer drugs [71]. Correspondingly, the loss of TAP2 expression is favorable for patients to respond to neoadjuvant chemotherapy [72]. Knox et al. [27] hypothesized that an SNP in the 3′UTR of *TAP2* interfered with miRNA binding, thus inhibiting the expression of the gene. In silico assays using PolymiRTS database 3.0 (http://compbio.uthsc.edu/miRSNP) (accessed on 10 October 2022) identified six common SNPs as several miRNA target site SNPs. To determine whether some of them could affect miRNA binding, the minimum free energy (MFE) was calculated for the common and variant alleles of each. The variant T allele of rs241456 significantly increased the MFE of binding for hsa-miR-1270 and hsa-miR-620 as compared with the common C allele. An earlier study discovered that *TAP2* mRNA levels negatively correlated with the expression of these miRNAs in brain tissues [41]. RNA electrophoretic mobility shift assays (RNA EMSA) showed that only hsa-miR-1270 bound to the T allele of rs241456 SNP in an allele-specific manner. The T allele was negatively correlated with the hsa-miR-1270 expression in kidney tissues, thereby confirming the biological relevance of miR-1270 in the regulation of *TAP2*. Luciferase reporter assay demonstrated that the expression of the T allele (but not C allele) harboring luciferase was efficiently suppressed by hsa-miR-1270 mimic as compared with the miRNA negative control in HEK 293 cells. These results help to explain the individual variability of drug metabolisms and the resistances to some drugs transported by TAP2 [27].

### 3.4. Transcription Regulators

The main expression regulators of DMET genes are the xenosensor transcription factors (TFs), such as aryl hydrocarbon receptor (AhR); constitutive androstane receptor (CAR, NR1I3); pregnane X receptor (PXR, NR1I2); peroxisome proliferator–activated receptor α (PPARα, NR1C1); retinoid X receptor α (RXR, NR2B1), the partner of CAR, PXR, and PPARα in heterodimerization; nuclear factor erythroid 2-related factor 2 (Nrf2, NFE2L2); and several other liver-enriched TFs [73,74,75].

#### 3.4.1. PXR

PXR is the best studied member of the nuclear receptor (NR) family in relation to the control of DMETs. PXR exerts its transcriptional regulation on CYP isoforms, such as CYP3A4 and CYP2B6; conjugation enzymes, such as UDP-glucuronosyltransferases and sulfotransferases; and transporters, such as multidrug-resistance protein 1 (MDR1) [76,77]. PXR is involved in human cancer by either directly affecting carcinogenesis or inducing drug–drug interactions and chemotherapy resistance [78,79,80]. So far, a large volume of data has been accumulated on the association between the SNPs in the 3′UTR of *NR1I2* and the treatment efficiency with different drugs. In particular, rs3732360 (C>T and C>G) and rs1054191 (G>A) have emerged to be associated with a change in doxorubicin pharmacokinetics via altering the miRNA-mediated posttranscriptional regulation of *PXR* in Indian breast cancer patients [81]. Earlier, rs3732360 and rs3732359 have been associated with a higher in vivo CYP3A activity (measured as midazolam clearance) in the African American subpopulation [82]. A later observation suggested the association of rs3732359 AA with worse survival prognosis in the women with breast cancer treated with FAC (doxorubicin, 5′-fluorouracil, and cyclophosphamide) chemotherapy [83]. A significant effect of rs3732359 on cyclosporine A dose-adjusted concentration was also demonstrated [84]. According to in silico data, all listed SNPs destroy/generate target sites for several miRNAs [38]. However, only PASSPORT-seq has been used for their experimental study. This approach showed that the C to T substitution (rs3732360), generating potential miR-3p/502-3p/500/502a binding sites, decreased expression of the reporter construct carrying the T allele in HepaRG cells in a statistically significant manner. On the other hand, rs1054191 and rs3732359 failed to exert any effect in any of the four cell lines used in this experiment [38].

#### 3.4.2. GATA4

GATA4 is a liver-specific TF that regulates the expression of different liver detoxifying enzymes and transporters [85]. Several studies have shown that GATA4 is strongly involved in the transcriptional activation of human *CYP2C9*, a major metabolizing enzyme in the warfarin pharmacological pathway [85]. One 3′UTR SNP, rs12458, located in *GATA4* with the potential to change miRNA-binding site, was genotyped in the study of 526 patients undergoing warfarin anticoagulation therapy using a simple PCR-based restriction fragment length polymorphism (PCR-RFLP) method. Bleeding is the most serious complication of warfarin anticoagulation therapy occurring even in the patients with therapeutic international normalized ratio (INR) range. Six following miRNAs were in silico predicted for *GATA4* rs12458: miR-556-5p, miR-4279, miR-500b, miR-502-5p, miR-526b, and miR-362-5p. The patients with the rs12458 AT (TT) genotypes of the *GATA4* gene had a lower risk of bleeding as compared with the patients carrying the wild-type AA genotype. The authors conclude that the mutant rs12458 T allele changes the *GATA4* expression and, as far as GATA4 regulates the expression of *CYP2C9* gene, also alters the warfarin metabolism (determined by CYP2C9 enzyme) and influences the correspondingly side effects [86]. A later study revealed that the rs12458T allele showed a considerably decreased activity in luciferase reporter assay, supporting a possible role of the rs12458 variant in the regulation of binding of certain miRNAs with the *GATA4* mRNA [87]. 

Most comprehensive examples of the SNPs affecting miRNA target sites in 3′UTR of pharmacogenes are summarized in the Table 1.

## 4. Variation in 3′UTR miRNA Target Sequences of the Genes Not Belonging to Conventional Drug-Metabolizing System

In addition to the SNPs in miRNA target sequences in the 3′UTRs of conventional genes from the drug-metabolizing system that are associated with individual susceptibility to pharmaceuticals, the examples of such variations in the genes not belonging to this system are known. The products of these genes can fulfill most diverse functions. Typically, these examples are found when purposefully searching for the SNPs in miRNA target sequences in the 3′UTRs of the genes/groups of genes in a certain way potentially associated with drug response. In particular, these genes may code for DNA repair enzymes, RNA editing enzymes, the enzymes involved in specific drug metabolism (including both drug activation and drug inactivation), and various regulatory proteins.

### 4.1. RPA1

The *RPA1* gene codes for replication protein A1, a protein of the nucleotide excision repair (NER) system, the activity of which may influence the response to platinum chemotherapy and the clinical outcomes of patients [88,89]. As has been shown, rs5030740, located in the 3′UTR of *RPA1*, is associated with the response to oxaliplatin chemotherapy [32]. According to eQTL analysis, the C allele of rs5030740 is significantly associated with higher *RPA1* mRNA expression levels. Using the SNPinfo server, the authors predict that rs5030740 may destroy or create binding sites for nine miRNAs. Among them, let-7e-5p is negatively correlated with the *RPA1* expression in colorectal tumors and adjacent normal tissues in the rs5030740 TT group but not in the rs5030740 C group. On the contrary, miRNA-625-5p is somewhat positively correlated with the expression of *RPA1* for the rs5030740 TT genotype. A comparison of the luciferase activities of *RPA1* 3′UTR constructs containing the rs5030740 C and T alleles in the presence of let-7e-5p or miR-625-5p has shown that the reporter gene expression is upregulated only in the case of cotransfection with the plasmid overexpressing let-7e-5p and the reporter construct carrying the C allele. This suggests that the C allele disrupts let-7e-5p binding site in the 3′UTR of *RPA1* gene. Thus, a low *RPA1* expression in the rs5030740 TT group increases the susceptibility to oxaliplatin in colon cancer cells and inhibits the proliferation after oxaliplatin treatment [32].

### 4.2. ADAR

Adenosine deaminase RNA specific (*ADAR*) gene belongs to adenosine-to-inosine (A-to-I) RNA editing system. The dysregulation of the genes constituting this system is involved in cancer development and drug resistance [90]. As has been shown, rs1127317 located in the *ADAR* 3′UTR is significantly associated with the survival rate of non-small cell lung cancer (NSCLC) patients and drug response, namely, an elevated ADAR expression and profoundly shortened survival after EGFR-TKI therapy was observed in the carriers of the rs1127317 C allele [33]. Examination of the clinical specimens has shown that the rs1127317 AC and CC genotypes are associated with the upregulation of *ADAR* gene expression as compared with the AA genotype in both NSCLC and normal lung tissue. According to miRNASNP-v3 predictions, rs1127317 influences gene expression by disturbing the interactions between *ADAR* mRNA and miR-454-5p, miR-340-5p, and miR-142-5p. Of them, only the cotransfection of NSCLC cells with miR-454-5p mimics and pGL3-ADAR-A allele reporter construct remarkably declined the luciferase activities as compared with the cotransfection using miR-454-5p mimics and pGL3-ADAR-C reporter construct. The authors infer that the C to A substitution creates a miR-454-5p binding site and decreases the level of *ADAR* mRNA. As a result, the patients carrying the rs1127317 C allele, which correlates with an elevated ADAR expression in tumors, have a considerably shortened survival after EGFR-TKI therapy as compared with the A allele carriers. In vitro, the silencing of *ADAR* essentially enhanced the gefitinib susceptibility of NSCLC cells [33].

### 4.3. CTNNBIP1

β-Catenin-interacting protein 1 (CTNNBIP1) is a regulatory protein. It is also known as Icat, an inhibitor of β-catenin. Its function consists in prevention of the formation of β-catenin complex with the TCF/LEF transcription factor, which inactivates the transcription of Wnt target genes, thereby negatively regulating the Wnt/β-catenin pathway [91]. CTNNBIP1 acts as a tumor suppressor in colorectal [92], cervical [93], and gastric [94] cancers. Correspondingly, it is regarded as a promising chemotherapeutic target. A study of the associations between the SNPs in *CTNNBIP1* gene and the platinum treatment response of the Han Chinese patients suffering from epithelial ovarian cancer discovered this association for rs935072, located in *CTNNBIP1* 3′UTR. It has been shown that the rs935072 AT/TT genotypes are associated with a decreased risk of developing chemoresistance as compared with the AA genotype. Using the online tools of MirSNP, TargetScan, and SNPInfo, the authors assume that miR-27a-3p is a potential miRNA able to bind to the 3′UTR of *CTNNBIP1* and that the rs935072 A>T substitution may affect this binding. This hypothesis is confirmed by luciferase reporter assay. Using both IGROV1 and OVCAR-8 cell lines, it has been shown that the transfection with miR-27a-3p mimic downregulates the luciferase activity in the case of the rs935072 A allele but not the T allele. Correspondingly, the authors hypothesize that the rs935072 A>T substitution affects the efficiency of microRNA-27a-3p binding and that a weak binding affinity of miR-27a-3p for the 3′UTR region may upregulate *CTNNBIP1* and decrease the risk of developing the resistance to platinum treatment. This conclusion is confirmed by the fact that an overexpression of CTNNBIP1 sensitizes ovarian cancer cells to platinum therapy [95].

In addition, the preliminary data for several other genes suggest the SNPs in their UTRs can regarded as potential regulators influencing the rate of transcriptional response to pharmaceuticals. Among these genes is the gene coding for carbonyl reductase 1 (CBR1), a key enzyme transforming nonsteroidal antiinflammatory drug loxoprofen into its pharmacologically active trans-OH metabolite [96]. The G>A substitution (rs9024) in the *CBR1* 3′UTR decreases the loxoprofen bioactivation in the human liver and in lymphoblastoid cell lines [97], which can be important for selecting individual doses of this drug. Another example is the *VKORC1* gene, coding for vitamin K epoxide reductase; this enzyme converts vitamin K epoxide to vitamin K. VKORC1 is a key enzyme for the physiological recycling of vitamin K, an important cofactor for a number of coagulation proteins [98]. Warfarin is an oral anticoagulant widely prescribed for prevention and treatment of thromboembolic events [99]. The allele A of rs7294 in the *VKORC1* 3′UTR is associated with higher warfarin dose requirements based on genome-wide association studies, meta-analysis, and population-based association studies [100]. According to predictions, the miR-133a-3p binding to the site in *VKORC1* 3′UTR, carrying this allele, is worse as compared with the wild-type G allele, which may cause a constitutive *VKORC1* mRNA expression and, correspondingly, the need in higher warfarin doses [100]. This list can be continued and the SNPs it comprises are promising candidates for the further functional studies.

## 5. Unbiased Genome-Wide Approaches Appropriate for Detecting the SNPs That Influence the Interaction of miRNAs with Their Target Sites and Associated with Individual Drug Response

The examples described in Section 3 and Section 4 demonstrate that mirSNPs located in the 3′UTRs of a wide range of genes (either directly associated with drug metabolism or not associated with it) may be involved in the individual response to a drug. That is why the formation of most comprehensive panels of such SNPs and the discovery of new target genes require the use of the experimental genome-wide approaches that avoid a particular initial hypothesis. Currently, three approaches meet this condition, namely, the search for allele-specific expression (ASE) or binding (ASB) events and eQTL analysis [7].

So far, the largest-scale studies implemented using these approaches and focused on the genome-wide detection of the polymorphisms “responding” to a drug are the search for transcriptome ASE data on the response to 50 compounds [101] and for eQTLs in the response to γ-interferon [102]. The primary (raw) data obtained in these studies allowed us to discover new SNPs influencing the interaction of miRNAs with their target sites and associated with individual response to drugs.

In particular, we selected the polymorphisms localized to the 3′UTRs of the known transcripts among the 253 ASE events either emerging or intensifying in five human cell types—lymphoblastoid cell line (LCL), primary peripheral blood mononuclear cells (PBMCs), human umbilical vein endothelial cells (HUVECs), smooth muscle cells (SMCs), and melanocytes—in response to exogenous compounds, including several pharmaceuticals [101]. Next, we searched for the potential miRNA binding sites affected by these polymorphisms using the PolymiRTS database [103]. In this process, only the polymorphism variants with their ASE manifestation and direction coinciding in all biological replicates were taken from the ASE collection. Since the initial publication does not specify a particular haplovariant with preferable expression, we determined this from the ratio of the initial read counts mapped on the variants.

**Figure 2:**
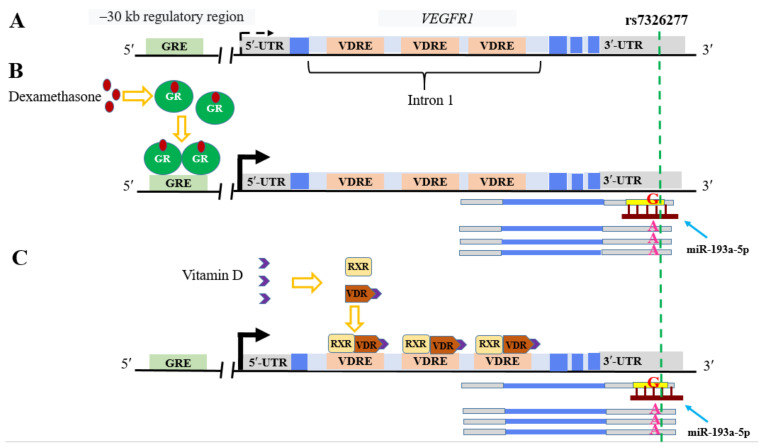
Putative mechanism underlying the effect of rs7326277 on the level of induced expression of *VEGFR1* gene. (**A**) Before the treatment with dexamethasone or vitamin D, the expression of this gene in a human primary cell line (HUVEC) is at a background level (dashed arrow); (**B**) dexamethasone activates the glucocorticoid receptor (GR) and the receptor interacts with the GRE in distant region of *VEGFR1* gene (ENCODE), activating transcription (solid arrow). The mRNA molecules carrying rs7326277 allele G bind miR-193a-5p in the MRE site in 3′UTR, decreasing the number of mRNA copies as a result of degradation. (**C**) The interaction of vitamin D (its active derivative) with its receptor (VDR) activates *VEGFR1* gene transcription (solid arrow) via the binding of VDR/RXR heterodimeric complex with the three VDRE sites in intron 1 (https://www.ncbi.nlm.nih.gov/geo/query/acc.cgi?acc=GSE145584) (accessed on 10 October 2022). Allelic imbalance is determined by the miR-193a-5p–mediated decrease in the number of the mRNA copies carrying rs7326277 allele G.

It has emerged that 58 of the 253 ASE events caused by the response to common drugs, steroid and peptide hormones, metal ions, dietary components, and environmental contaminants [101] coincide with the variations affecting the miRNA binding sites in the 3′UTRs of different mRNAs. In particular, five ASE events are associated with the response to dexamethasone, widely used in the therapy of autoimmune and allergic diseases, several cancer diseases, and, recently, COVID-19 [104]. Currently, rs7326277 seems the most interesting of them. According to our analysis, the A to G substitution (rs7326277) generates the miR-193a-5p binding site in the 3′UTR of the *VEGFR1* (*FLT1*) gene, an important player in tumor progression, inflammation, and epithelial–mesenchymal transition [105,106]. According to Moyerbrailean et al. [101], the expression of this gene in the primary endothelial cells (HUVECs) before the dexamethasone treatment is at the background level. The gene is induced by glucocorticoids; note that the induction level of its mRNA transcribed from the chromosome carrying the G allele is considerably lower as compared with the chromosome carrying the A allele. Noteworthy that the treatment of HUVECs with vitamin D, displaying protective and therapeutic effects in autoimmune (multiple sclerosis, lupus erythematosus, and rheumatoid arthritis), infectious (tuberculosis and acute respiratory viral infections, including COVID-19), cardiovascular, and cancer diseases gave identical results [107,108,109]. The *VEGFR1* gene is also induced by this agent; interestingly, the induction level of the mRNA transcribed from the chromosome carrying the G allele was also considerably lower as compared with the chromosome carrying the A allele. It is known that the carriers of rs7326277 (GA + GG) variant display a considerably lower risk of both chronic obstructive lung disease and lung cancer as compared with the carriers of AA genotype. The expression of *VEGFR1* gene in the lung and tumor tissues is also significantly lower in the carriers of the G variant [110]. Thus, rs7326277 via its impact on the miR-193a-5p affinity for its target site in the mRNA 3′UTR of *VEGFR1* modulates the response of this gene to glucocorticoid hormones and vitamin D (Figure 2), thereby contributing to the formation of predisposition to a number of diseases and individual susceptibility to the corresponding therapy.

**Figure 3:**
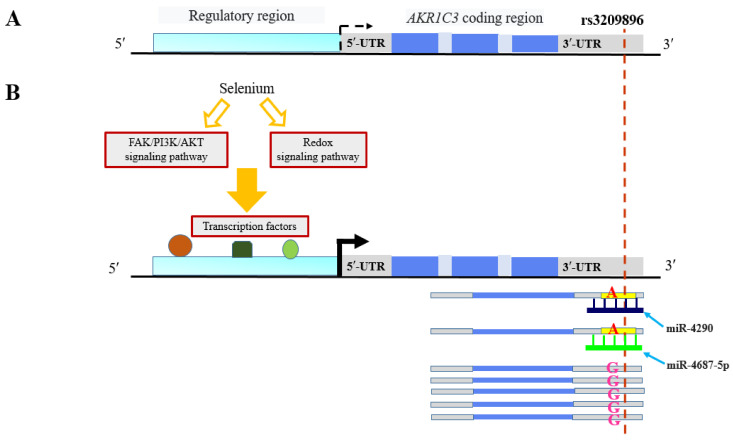
Putative mechanism underlying the effect of rs3209896 from the 3′UTR of *AKR1C3* gene. (**A**) Before the treatment with selenium-containing preparations, the expression of this gene in a human primary cell line (HUVEC) is at a background level (dashed arrow); (**B**) selenium preparations can activate either FAK/PI3K/AKT or redox signaling pathway, resulting in binding of transcription factors with their sites in the *AKR1C3* regulatory region, and triggers transcription (solid arrow). The mRNA molecules carrying rs3209896 allele A bind miR-4290 or miR-4687-5p in the MRE site of 3′UTR, thereby decreasing the mRNA copy number as a result of degradation.

Another interesting example is the rs3209896 from the 3′UTR of *AKR1C3* gene. According to our data, the G to A substitution generates miR-4290 and miR-4687-5p binding sites versus the data by Moyerbrailean et al. [101], who assume that this decreases the response of *AKR1C3* gene in HUVECs to selenium treatment. The selenium induction of this gene can be determined by either the activation of FAK/PI3K/AKT or redox signaling pathways (Figure 3) [111,112], while the binding of miR-4290 or miR-4687-5p with the A allele decreases the response to the preparation (Figure 3). As is known, selenium-containing preparations display immunomodulatory properties as well as increase the cytotoxic activity of chemotherapeutics and enhance the overcoming of tumor drug resistance [113]. AKR1C3 is involved in the regulation of metabolisms of prostaglandins, steroids, and retinoids as well as in the processes of tumor progression [114], suggesting a likely role of the detected mechanism of the change in selenium susceptibility in these processes. To a certain degree, the data on a decrease in the recurrence-free survival of the breast cancer patients heterozygous in rs3209896 confirm this assumption [83].

In a similar manner, we have found 307 SNPs among the 5270 SNPs behaving as eQTLs that provide the response of PBMCs to interferon-γ [102] located in the predicted miRNA binding sites the calculated affinity of which for at least one miRNA would change as a result of a nucleotide substitution. The samples of 5270 and 307 SNPs do not show any statistically significant differences in GO/KEGG enrichment terms, suggesting that they belong to the same functional group. The highest enrichment for the sample of 307 SNPs was shown for the terms “cellular response to stress” (41 gene, FDR = 3.71 × 10^−8^) and “immune response” (36 genes, FDR = 5.59 × 10^−6^). According to mSigDB data, the highest enrichment (44 genes, FDR = 1.66 × 10^−11^) was demonstrated for the genes induced by the FluMist influenza virus live vaccine in PBMCs. In addition, this sample emerged to be enriched with the target genes for miR-8485 (28 genes, FDR = 4.3 × 10^−7^), confirming the role of this miRNA in the regulation of immune response, which was revealed by analyzing the network of interactions between long noncoding RNAs, miRNAs, and mRNAs in heart failure [115].

It is important to keep in mind that in the case of ASE identification and the more so in the case of the search for eQTLs, part of the SNPs in the formed sample will be represented by marker variants rather than regulatory ones [7]. That is why additional experimental studies, for example, PASSPORT-seq, are necessary to extract the SNPs actually influencing the interaction of miRNAs with their target sites. Note also that the ASE approach has an essential advantage as compared with eQTL analysis, which consists in a considerable reduction in the number of analyzed samples from many tens and even hundreds in eQTL search to several or even a single sample in ASE [7,116]. Thus, ASE approach gives the opportunity to considerably increase the number of temporal and environmental conditions that can be analyzed in parallel, thereby providing unique possibilities for the studies in pharmacogenetics and pharmacogenomics.

We cannot but mention the CLIP technology [44] among the other unbiased genome-wide approaches. Theoretically, this approach is also appropriate for a high-throughput recording of induced ASB events and, consequently, for the search for new SNPs affecting the interaction of miRNAs with their target sites and associated with the individual response to a particular drug provided that the patterns of AGO–RNA binding induced by this drug. However, this approach is the most expensive and laborious of all considered here.

## 6. Conclusions

The miRNAs are an important class of gene expression regulators acting at a posttranscriptional level via translational repression or mRNA degradation [13,14,15]. Correspondingly, the SNPs both in seed sequences of miRNAs and their target sequences in mRNA 3′UTRs may be of functional significance (rSNPs). A large volume of the data on rSNPs in the mRNA 3′UTRs of the genes associated with various diseases has been accumulated so far and systematized in a number of reviews [11,12,26]. In contrast, the data on the variations that can be used to assess the efficiency of therapies with different pharmaceuticals are still rather few and disorganized. As far as we know, our review is the first attempt to fill this gap.

First and foremost, we have succeeded in collecting a number of examples of the rSNPs located in the 3′UTRs of conventional genes from the drug-metabolizing system that are important contributors to the determination of therapeutic efficiency of different pharmaceuticals depending on their alleles. Far from all genes of this system are included in this list; this is primarily determined by laboriousness of the gene-by-gene studies of rSNPs and, as a consequence, a slow accumulation of the corresponding experimental data. An efficient high-throughput approach to detection of 3′UTR rSNPs is the MPRA-based PASSPORT-seq [38]. However, this approach has been so far used for concurrent testing of only 100 SNPs located in the 3′UTRs of known pharmacogenes [38,44], which in no way covers the overall set of potential rSNPs in these regions and the total set of pharmacogenes. Currently, the performance of this method can be considerably increased thanks to massively parallel synthesis, which makes it possible to increase the size of MPRA libraries to tens of thousands of simultaneously tested variants [117,118]. However, an important limitation of the MPRA-based approaches is still the need to use a large number of different cell lines to obtain the most complete result due to cell line–specific function of mirSNPs [38].

Analysis of the available data of gene-by-gene studies has allowed us to find the examples of SNPs in miRNA target sequences in 3′UTRs of the genes not belonging to the conventional drug-metabolizing system that are associated with individual susceptibility to pharmaceuticals. The products of these genes can fulfill most different functions, such as involvement in DNA repair, RNA editing, specific drug metabolism (including both drug activation and drug inactivation), and transcription regulation. These findings pose the question on application of unbiased experimental genome-wide approaches that avoid a particular initial hypothesis in the search for potential rSNPs in miRNA target sequences. Theoretically, three approaches meet this condition, namely, the search for allele-specific expression (ASE) or binding (ASB) events and eQTL analysis [7] although none has been so far purposefully used to resolve this problem. Nonetheless, our analysis of the ASE data in the transcriptomes of five human cell lines before and after the treatment with 50 different compounds, including several pharmaceuticals [101], discovered clinically significant rSNPs in miRNA target sequences able to determine the individual response to dexamethasone, vitamin D, and selenium. In general, the facts that transcriptome studies are the most available and inexpensive genome-wide approaches and the identification of ASE events requires a minimum number of biological samples [116] suggest that ASE approach is the most promising unbiased approach to search for the SNPs in miRNA target sequences that are associated with the individual susceptibility to pharmaceuticals.

## Figures and Tables

**Table 1:** Most comprehensive examples of the SNPs affecting miRNA target sites in 3′UTR of pharmacogenes.

mirSNPs/Gene/miRNA	In Silico Approaches	In Vitro Gene-by-Gene Approaches	PASSPORT-Seq	Drug Metabolisms and Drug Resistance	References
Neighboring rs2480256 and rs2480257/CYP2E1/miR-570	MicroSNiPer computational service	Transient and stable luciferase reporter assay with miRNA cotransfection	Support	Associated with the trabectedin-induced hepatotoxicity in patients with advanced soft tissue sarcoma	[36,50]
rs15524/CYP3A5/hsa-miR-500a	MirSNP database search, SNPinfo	Transient luciferase reporter assay with miRNA cotransfection	Support	Associated with the tacrolimus serum concentration in myasthenia gravis therapy and sirolimus serum concentration of kidney-transplanted patients	[51,52,53]
rs70950385 = combination of rs70950385 and rs12979898/*CYP2B6*/miR-1275	Analysis using different algorithms and databases	Luciferase reporter assays	Support	Associated with antiretroviral drug efavirenz hydroxylation activity in human liver microsomes and efavirenz pharmacokinetics	[54]
rs3181842/CYP2B6/miR-1581/4537	TargetScan, SomamiR database	Luciferase transfection into HepG2 and LO2 cells	Support	Influence on the propofol target plasma concentration in patients under total intravenous anesthesia	[30,55]
rs6839 and rs1042157/SULT1A1/miR-631	MicroInspector and RNAhybrid programs MFE calculation	Luciferase reporter assay with miRNA cotransfection, decrease in miR-631 level with inhibitor	Support	Associated with transformation of active metabolite 4-hydroxy-tamoxifen into inactive 4-hydroxy-tamoxifen sulfate in cancer treatment	[28,63]
rs8330/UGT1A/miR-1286 rs10929303/UGT1A/miR-21-3p, miR-141-3p, and miR-200a-3p	miRanda and RNAhybrid programs	Luciferase reporter assays with cotransfection of 2048 mimic miRNA library	Support	Influence on analgesic drug acetaminophen glucuronidation status in healthy subjects, gefitinib plasma concentration, and gefitinib-induced rash in cancer patients	[35,67,69]
rs241456/ABCB3/hsa-miR-1270	PolymiRTS database, MFE calculation	RNA EMSA, luciferase reporter assay with miRNA cotransfection	ND	Associated with response to neoadjuvant chemotherapy in cancer patients	[27,72]
rs12458/GATA4/miR-556-5p, miR-4279, miR-500b, miR-502-5p, miR-526b, and miR-362-5p	In silico prediction (not detailed)	Luciferase reporter assay	ND	Associated with bleeding complication of warfarin anticoagulation therapy	[86,87]
